# Modelling and genetic dissection of staygreen under heat stress

**DOI:** 10.1007/s00122-016-2757-4

**Published:** 2016-08-22

**Authors:** R. Suzuky Pinto, Marta S. Lopes, Nicholas C. Collins, Matthew P. Reynolds

**Affiliations:** 1 International Maize and Wheat Improvement Center (CIMMYT,Int.), Apdo. Postal 6-641, 06600 México, D.F., Mexico; 2 Australian Centre for Plant and Functional Genomics (ACPFG), School of Agriculture Food and Wine, University of Adelaide, Glen Osmond, Adelaide, SA 5064 Australia

## Abstract

***Key message*:**

**Staygreen traits are associated with heat tolerance in bread wheat. QTL for staygreen and related traits were identified across the genome co-located with agronomic and physiological traits associated to plant performance under heat stress.**

**Abstract:**

Plant chlorophyll retention—staygreen—is considered a valuable trait under heat stress. Five experiments with the Seri/Babax wheat mapping population were sown in Mexico under hot-irrigated environments. Normalized difference vegetation index (NDVI) during plant growth was measured regularly and modelled to capture the dynamics of plant greenness decay, including staygreen (Stg) at physiological maturity which was estimated by regression of NDVI during grainfilling. The rate of senescence, the percentage of plant greenness decay, and the area under the curve were also estimated based on NDVI measurements. While Stg and the best fitted curve were highly environment dependent, both traits showed strong (positive for Stg) correlations with yield, grainfilling rates, and extended grainfilling periods, while associations with kernel number and kernel weight were weak. Stg expression was largely dependent on rate of senescence which was related to the pattern of the greenness decay curve and the initial NDVI. QTL analyses revealed a total of 44 loci across environments linked to Stg and related traits, distributed across the genome, with the strongest and most repeatable effects detected on chromosomes 1B, 2A, 2B, 4A, 4B and 7D. Of these, some were common with regions controlling phenology but independent regions were also identified. The co-location of QTL for Stg and performance traits in this study confirms that the staygreen phenotype is a useful trait for productivity enhancement in hot-irrigated environments.

**Electronic supplementary material:**

The online version of this article (doi:10.1007/s00122-016-2757-4) contains supplementary material, which is available to authorized users.

## Introduction

The staygreen attribute, defined as “heritable delayed foliar senescence” (Thomas and Stoddart 1975) is considered as a selection criterion for crop improvement to extend grainfilling duration and ensure that grain size is not limited by lack of post-anthess assimilates. For many years the staygreen character has been empirically included in visual selection of breeding lines (Thomas and Ougham 2014) but its genetic basis is not well understood.

The visible symptom of a staygreen phenotype is the persistence of greenness, which actually represents only one of many processes involved in delayed leaf senescence. The permanence of the pigment can be due to disabled chlorophyll catabolism or modification of the chlorophyll *b* and chlorophyll *a* ratio (Thomas and Howarth 2000). Complex hormonal controls are involved in leaf senescence, where cytokinins are the main inhibitors; plant treatment with cytokinins has resulted in staygreen phenotypes of tobacco and *Arabidopsis* (Gan and Amasino 1995). Five types of staygreen have been distinguished (Thomas and Howarth 2000), which broadly can be grouped as cosmetic staygreen or functional staygreen. As their names indicate, in the first type of staygreen the tissue looks green even when photosynthetic activity has been decreased or stopped in contrast to the functional staygreen (Thomas and Ougham 2014). The latter is obviously the target of plant breeding. Staygreen has been associated with drought and heat tolerance (Kumari et al. 2007); for example in sorghum, grain yield is positively associated with staygreen under water limited conditions (Rosenow et al. 1983; Borrell and Douglas 1996). Similarly to drought environments, under heat stressed conditions the staygreen attribute seems to be advantageous. Genotypes that exhibit delayed loss of greenness after anthesis show superior agronomic performance (Kumari et al. 2007; Borrell and Douglas 1996; Borrell et al. 2000). The latter is because staygreen indicates higher photosynthetic assimilation in the late stages of plant development which contributes to increase crop yield; the reason can be an extended photosynthetic active phase or higher photosynthetic rate due greater retention of leaf nitrogen content (Harris et al. 2007). However, it is not yet clear if the physiological and genetic basis for delayed loss of greenness under heat are similar to drought. Mechanisms related to the staygreen phenotype conferring heat adaption may be for example, the conservation of nitrogen through reduction of plant size (including leaves, stems and roots) and modification of water uptake patterns as found under water limited conditions (Borrell et al. 2014a; Mace et al. 2012), but this needs to be confirmed. Sorghum plant with reduced leaf size and decreased tillering have proven to result in genotypes using a conservative strategy to reduce the use of soil water before anthesis for use during grainfilling when water is a limitation. Apparently the staygreen genes affect the expression of genes controlling hormones influencing plant growth (Borrell et al. 2014a). Neverthless, sorghum has shown correlations between staygreen and yield in environments yielding >6 t ha^−1^ (Jordan et al. 2012).

Genetic variability for staygreen has been identified and exploited in maize, oat, rice, wheat, fescue, soybean, pea, tomato, pepper, fruits, trees and other species (Barry et al. 2008; Armstead et al. 2006; Duvick et al. 2004; Thomas and Smart 1993; Thomas and Stoddart 1975). A number of studies have modelled the staygreen attribute as an indicator of photosynthetic activity. Deeper understanding of the dynamics and mechanisms affecting staygreen under high temperature environments are required to successfully exploit this attribute and improve plant adaptation to heat stress. Modelling canopy greenness dynamics over the whole crop cycle can help with this, while having obvious application in determining the best time for screening by identifying at what growth stage(s) differences in greenness are best associated with yield and show the best resolution. The factors affecting staygreen under high temperature conditions are unclear but a better understanding of canopy greenness dynamics are expected to (a) provide information about canopy activity at different time-points during the crop cycle which may be under independent genetic control, and (b) demonstrate when differences in greenness are best expressed in order to refine screening protocols.

Elevated temperatures and high irradiance promote the generation of reactive oxygen (ROS) species which can lead to cell damage and further accelerate loss of green biomass (McDonald and Vanlerberghe 2004; Christiansen 1978). In this regard, it seems that the staygreen genotypes have the ability to cope with the negative effect of heat stress either by minimizing the production and accumulation of ROS through the pigments such as xanthophylls and carotenes that protect the chloroplasts by dissipating excess of radiation energy, reducing damage to the photosynthetic apparatus (Hopkins and Hüner 2009; Suzuki and Mittler 2006; Zhao and Tan 2005). It is interesting that staygreen is frequently reported for leaf greenness while other organs that also contribute to total plant photosynthesis such stems and spikes are not always considered. CO_2_ absorbed by spikes represents at least 20 % of flag leaf CO_2_ captured in wheat (Teare et al. 1972) and estimates indicate that the spikes’ contribution to grain yield is variable depending of the conditions but can reach up to the 70 % in wheat and barley grown under stress (Maydup et al. 2010; Araus et al. 1993; Biscoe et al. 1973; Thorne 1963). Accurate quantification of individual leaf greenness (Harris et al. 2007) can be performed with the SPAD meter, and visual scoring, though more subjective, has been used to estimate greenness for decades (Kumar et al. 2010). The GreenSeeker spectral sensor offers an integrative high throughput approach to precision quantification of staygreen; it measures total canopy variation in green area including leaves, stems and spikes and permits screening of a large number of samples in a relatively short time (Lopes and Reynolds 2012); this enables potential application in large scale phenotyping including for QTL mapping. The current study applies this novel methodology measuring normalized difference vegetative index (NDVI) during the crop cycle so that the pattern of greenness decline could be determined. A number of NDVI-based staygreen related traits can be derived to enhance understanding of the mechanisms affecting plant’s greenness persistence; these include the proportion of plant greenness lost mid grainfilling (Gdecay); the estimation of the velocity of greenness loss (RS) which together with the type of NDVI curve can provide information about how fast are lost the plant greenness and photosynthetic activity; and the estimate of total green biomass (StgAUC and TotalAUC), parameters determining light interception. It is hypothesized that StgAUC and TotalAUC can reflect the accumulated plant greenness during a given period of time and that high values for these two traits are favorable for plant performance due to an increase in plant’s green area available for capturing radiation (Cossani and Reynolds 2012). The quantification of the staygreen attribute and other related traits in a wheat mapping population allows the identification of genetic loci controlling staygreen which can provide the tools to enable MAS to accelerate and improve efficiency of plant breeding. QTL mapping for staygreen has been performed for several species including *Lolium* (Thorogood et al. 1999), pearl millet (Howarth et al. 1994), wheat (Kumar et al. 2010; Vijayalakshmi et al. 2010), maize (Zheng et al. 2009) and sorghum (Harris et al. 2007; Tao et al. 2000).

It has been estimated that wheat yield is reduced 3–5 % per 1 °C increased above 15 °C during the grainfilling period (Gibson and Paulsen 1999). High temperatures result in accelerated plant growth, reduced plant size and shortened cycle, limiting the amount of light intercepted. In that sense, extending the grainfilling duration through delayed greenness loss seems to be especially advantageous in heat stressed environment. The exact profile of the staygreen attribute as a heat adaptive-trait still needs to be clarified but in the current study it is proposed that plant greenness during grainfilling is lost following different patterns and that these patterns can be modelled following linear and non-linear regression models. Finally it is anticipated that genotypic differences for the Stg trait and related parameters exist and that this trait can be mapped for QTL to provide new avenues in the understanding of mechanisms controlling plant staygreen and its association with yield and other physiological traits.

The specific objectives of this study were (1) to model plant senescence patterns of Seri/Babax RIL grown under heat-stressed, irrigated conditions, (2) to calculate a measure of staygreen (Stg) at physiological maturity using a linear regression model, and (3) to identify QTL linked to this character and additional traits associated with heat tolerance.

## Methods

### Germplasm and field experiment conditions

The population consisted of 167 RIL derived from crosses between two of CIMMYT's elite lines: Seri M82 (herein called Seri) derived from a ‘Veery’ cross (KVZ/BUHO//KAL/BB) and a sister line of the elite variety Baviacora M92 ‘Babax’ (BOW/NAC//VEE/3/BJY/COC). Both parents exhibit drought tolerance and high yield potential (Olivares-Villegas et al. 2007) while the population is characterized by a restricted range of height and phenology and does not segregate for major height, vernalization or photoperiod response genes (Pinto et al. 2010).

Five heat-stressed, irrigated trials were conducted during the seasons 2005, 2006, 2010, 2011 and 2013 in the Yaqui Valley, Northwest México; the site is a high radiation, irrigated environment. In 2005, 2006 and 2010 the trials were sown in February and in 2011 and 2013 the trials were sown in March. Based on the mean temperature at particular developmental stages, the trials were classified as: moderately hot (M), hot (H) or intensely hot (I) and are named with these letters followed by the last two digits of the sowing and harvest year (Table 1). Field experiments consisted of plots of one raised bed of 80 × 100 cm with two rows per bed; all the experiments were sown in two-replicate alpha-lattice designs. Sowing seed density was 15 gm^−2^ in the February and March trials. All trials were fully irrigated when ~50 % of available soil moisture was depleted in the 0–1 m soil profile.

**Table 1:** Average daily temperatures (°C), total evapotranspiration (Eto, mm) and total rain (mm) recorded during the vegetative, reproductive and grainfilling stages for the five Seri/Babax trials grown between 2005–2013 under heat-stressed, irrigated conditions in the Yaqui Valley in Northwest, Mexico

Environment	Year of sowing and harvest	Month of sowing	Heat stress intensity	Measurements	Days to heading (dae)	GF length (days)	Maximum^a^ 3 days (°C)	Stage	Daily air temperature (°C)			Rain (mm)	Eto (mm)
									Maximum^b^	Minimum^b^	Mean^b^		
M10	2010	February	Moderate (M)	Stg rel traits Agr and Phys traits	53	31	40.0	Emergence to heading −10 days Heading ±10 days Heading +10 days to maturity	28.8 30.5 37.0	8.8 11.7 12.5	18.8 21.1 24.7	0 0 0	160 119 174
H05	2005	February	Hot (H)	Stg rel traits Agr and Phys traits	53	27	37.2	Emergence to heading −10 days Heading ±10 days Heading +10 days to maturity	31.8 34.7 35.5	10.0 12.3 19.0	20.9 23.5 27.2	0 0 0.70	231 141 103
H11	2011	March	Hot (H)	Agr and Phys traits	50	26	39.6	Emergence to heading −10 days Heading ±10 days Heading +10 days to maturity	33.4 35.1 38.4	12.6 12.7 18.5	23.0 23.9 28.5	0 0 0	235 169 119
I06	2006	February	Intense (I)	Agr and Phys traits	55	28	42.4	Emergence to heading −10 days Heading ±10 days Heading +10 days to maturity	33.7 38.4 39.5	10.8 15.9 20.8	22.3 27.1 30.2	0 0 0	233 154 158
I13	2013	March	Intense (I)	Stg rel traits Agr and Phys traits	49	25	38.9	Emergence to heading −10 days Heading ±10 days Heading +10 days to maturity	33.1 36.7 37.0	12.5 15.4 22.2	22.8 26.1 29.6	0 0 0.3	243 167 138

Non stressed environments are regularly sown during November–December where daily maximum temperatures of the anthesis stage for wheat crop are commonly <30 °C, season mean is 17.7 °C and ranging between 5.50 and 31.0 °C. Trials are named with letters M (moderately hot), H (hot) or I (intensely hot) followed by the last two digits of the sowing and harvest year

*dae* days after emergence, *GF* grainfilling, *Eto* evapotranspiration, *Agr and Phys* agronomic and physiological

^a^Maximum average of 3 days across the whole plant cycle

^b^Average of the daily maximum/minimum/mean temperature recorded during the days comprised in the specified period

### Phenotyping

Physiological and agronomical traits were recorded in the five trials according to standard procedures detailed elsewhere (Reynolds et al. 2001). These included: repeated measurements during the vegetative (v) and grainfilling stages (g) for the normalized difference vegetation index (NDVI), flag leaf chlorophyll (Chl) and canopy temperature (CT); individual measurements were averaged for these traits and a single value is presented. Also recorded were the number of days to reach heading (heading) and physiological maturity (maturity), plant height (height), grain yield, kernel number (KN), grain weight (TGW) and the grainfilling rate [GFR = yield/(days to maturity − days to heading)]. NDVI was measured by canopy reflectance with a GreenSeeker (Optical Sensor Unit, 2002 NTech Industries, Inc., Ukiah, CA, USA). The chlorophyll of the flag leaf was assessed using a portable chlorophyll meter (SPAD-502 Minolta, Spectrum Technologies Inc., Plainfield, IL, USA) and the CT was recorded using an infrared thermometer (Mikron M90 series) 2–3 times per week avoiding cloudy and windy days according to the protocol described in Reynolds et al. (2001).

### Estimation of staygreen related traits

**Fig. 1:**
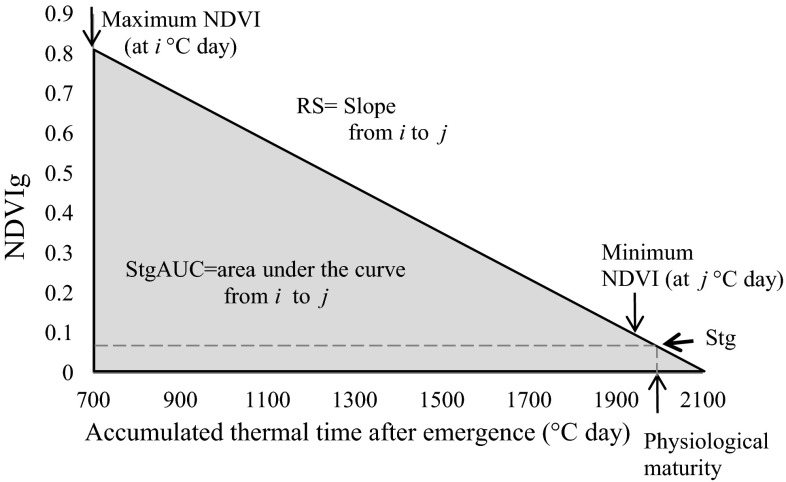
Diagram illustrating calculation of staygreen traits, rate of senescence (RS), StgAUC (area under the curve during the NDVIg decline phase) and Stg (greenness at physiological maturity). NDVIg: normalized difference vegetative index during grainfilling *i* thermal time with maximum NDVIg, *j* thermal time with minimum recorded NDVIg; RS was calculated as the linear slope from *i* to *j* for all the genotypes, *Stg* staygreen, residual greenness remaining at physiological maturity calculated using a linear regression for each genotypes

Staygreen (Stg) was calculated using linear regression analyses of NDVI readings from heading until shortly after maturity according to Lopes and Reynolds (2012), given that anthesis under heat stress occurs very shortly after heading. The regression equation for each experimental plot was obtained by plotting NDVI during grain filling (NDVIg) against days after heading; Stg was calculated by substituting the maturity day in the equation. Stg is a unitless trait given that it is based on a NDVI ratio. The rate of senescence (RS) for each genotype was calculated from the slope of the NDVIg decline against thermal time (°C) using a linear regression equation (Fig. 1). Greenness decay (Gdecay) was calculated as the percentage of NDVI decline in the first half of the grainfilling stage (in number of days after heading). Staygreen-area (StgAUC) and Total area (TotalAUC) were calculated as the area under the curve with starting points at maximum NDVI (for StgAUC) or at crop establishment (TotalAUC) and using the corresponding thermal time for each case. Stg and staygreen related traits (RS, Gdecay, StgAUC, TotalAUC) were estimated only in three environments: M10, H05 and I13, due to insufficient NDVI data in H11 and I06.

### Modelling NDVI along the crop cycle and during the grainfilling period

The modelling of NDVI curves across the crop development period and during staygreen decay in the grainfilling phase were performed in R 3.1.0 (http://www.R-project.org/) applying a sigmoidal function. In the M10 environment NDVIv (NDVI during the vegetative stage) was not recorded before 500 degree-days (dd, °C d) but in order to draw an NDVI trend for the whole cycle, this gap was filled using NDVI from H05 trial, given that comparable values were expected because NDVI for both trials performed similarly after 500 dd. (dotted line, Fig. 4). This assumption had no effect on the calculated Stg values or Stg related traits, except on TotalAUC, since only the later included these inferred NDVI values. For this analysis, a non-linear model was developed by combining two sigmoidal functions as given by the following equation:

$${\text{N}}\widehat{\text{DVI}}_{\text{TT}} = \frac{{{\text{NDVI}}_{\hbox{max} } }}{{1 + e^{{ - r_{\exp } ({\text{TT}} - i_{\exp )} }} }}\left( {1 - \frac{1}{{1 + e^{{ - r_{sen} ({\text{TT}} - i_{sen)} }} }}} \right),$$

where TT is the thermal time (i.e. °C days), is the simulated NDVI at TT, NDVI_max_ is the season maximum NDVI parameter, *r*_exp_ is a canopy expansion rate parameter, *i*_exp_ is a canopy expansion inflection point parameter, *r*_sen_ is a canopy senescence rate parameter, and *i*_sen_ is the inflection point of canopy senescence. Each genotype was individually modelled for NDVIg after heading following linear and non-linear models using the equations:

Linear model:

$${\text{NDVI}}_{\text{TT}} = m{\text{TT}} + b {\text{Curve type 1}}.$$

Non-linear models:

$${\text{NDVI}}_{\text{TT}} = - a{\text{TT}}^{2} + b{\text{TT}} + c {\text{Curve type 2}},$$

$${\text{NDVI}}_{\text{TT}} = a{\text{TT}}^{2} + b{\text{TT}} + c {\text{Curve type 3}}.$$

The best fitted model was selected based in the Bayesian information criterion (BIC).

### Statistical and QTL mapping analyses

Adjusted means were obtained in SAS v9.0 using ANOVA mixed models to obtain the best linear unbiased prediction (BLUPs); spatial adjustment was included in the analysis by adding the effect of row and column according to the location of each plot in the field. Pearson’s phenotypic correlations (*r*_*P*_) were calculated using the formula of Roff (1995) from the adjusted means. The QTL mapping analyses were performed in GenStat 15th edition in a Composite Interval mapping procedure using a threshold LOD value of 2 to identify all QTL candidates and LOD > 3.5 for defining consistent QTL. QTL mapping was performed individually by trial and by trait, and also for each trait combined across environments.

The Seri/Babax population map used here in was previously constructed and consisted of 475 markers: 118 SSR (Single Sequence Repeat), 212 AFLP (Amplified Fragment Length Polymorphism), and 145 DArT (Diversity Array Technology) markers distributed over 20 chromosomes, only the chromosome 3D is missing (McIntyre et al. 2010). Previous QTL mapping studies have been reported using earlier versions of this map (Pinto et al. 2010; Lopes and Reynolds 2012).

## Results

### Analysis of agronomic and physiological traits

**Fig. 2:**
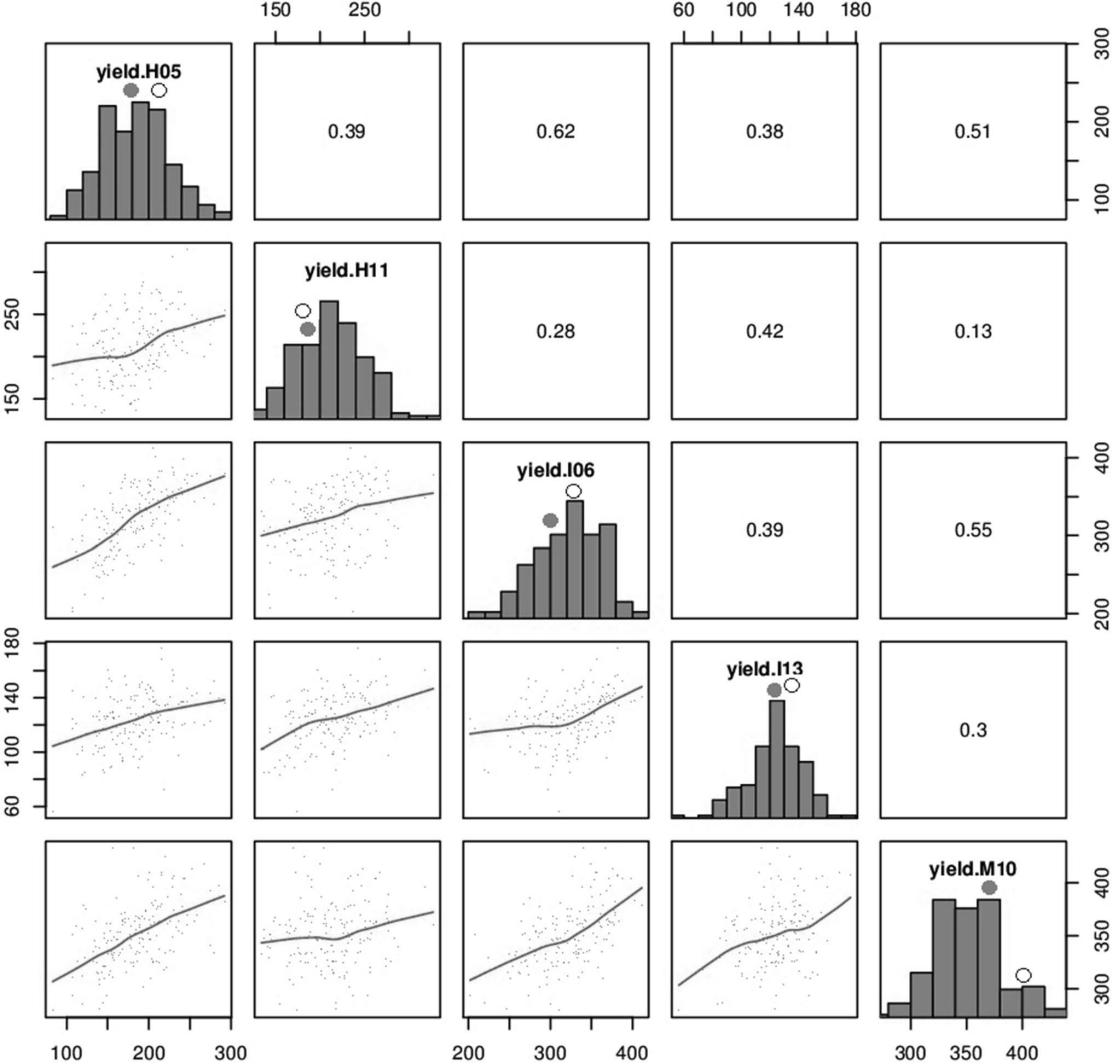
Associations of yield in the Seri/Babax population across five heat-stressed, irrigated environments grown between 2005 and 2013. The *diagonal* contains the yield histogram for each environment, the *lower diagonal* a *scatter plot* and loess smoothing line between all environments, and the *upper diagonal* shows the phenotypic correlations (*r*_*P*_). *r*_*P*_ > 0.15 are significant at *p* = 0.05; *r*_*P*_ > 0.19 are significant at *p* = 0.01; *r*_*P*_ > 0.24 are significant at *p* = 0.001. In the histograms Seri is represented with a *filled circle* and Babax with an *empty circle*

**Fig. 3:**
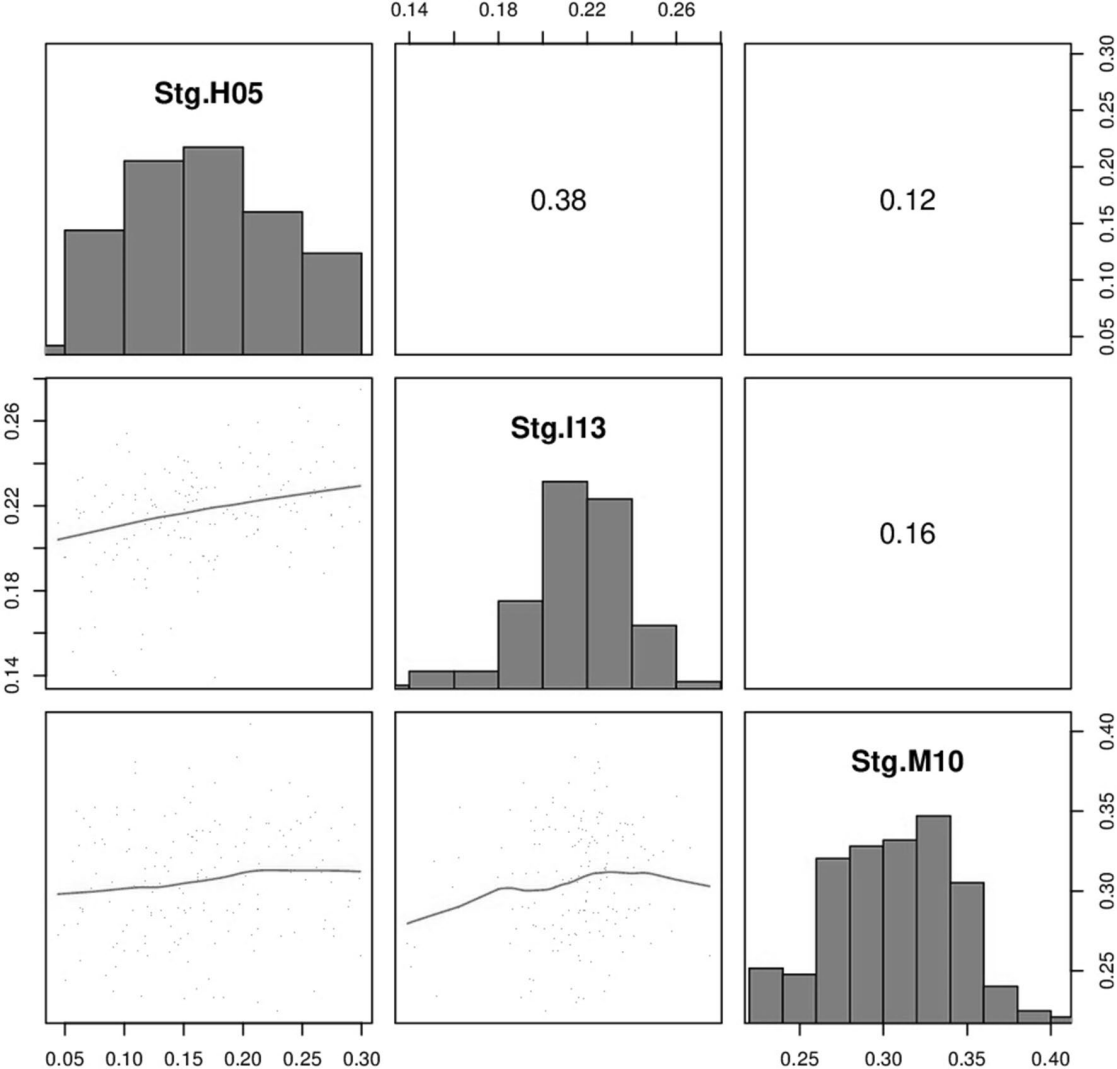
Stg correlations for the Seri/Babax population across three heat-stressed, irrigated environments sown between 2005 and 2013. The *diagonal* contains the Stg histogram for each environment, the *lower diagonal* a *scatter plot* and loess smoothing line between environments, and the *upper diagonal* shows the phenotypic correlations (*r*_*P*_)

The adjusted means and basic statistics for all traits calculated across the four trials for parents and RILs are presented on Table 2. The two parents showed similar expression for Stg, phenology and other traits while a much wider range was observed in the RIL. The rate of senescence (RS) for both parents averaged across environments indicated that the NDVIg decreased by about 8 SPAD-units each degree day (°C), similarly to the estimated population mean. Gdecay across environments ranged from 18 to 44 % and averaged 31.2 % for the RILs. Heading time was found to be relatively constant across parents and RILs, with a range of 13 days observed across environments. Pearson’s correlations showed that trial associations were positive and significant for yield (Fig. 2). Staygreen (Stg) was found to not well associated (*p* > 0.05) across the three environments (Fig. 3) varying from 0.12 to 0.38 but Stg showed consistent and positive correlation with kernel number (KN), thousand grain weight (TGW) and yield (Supplementary Fig. 1). The correlation between Stg and TGW was the weakest on average (Table 3), although it was still significant (*p* < 0.05). The distribution of the Stg trait showed that it varied across environments, ranging from 0.2 to 0.4, 0.05 to 0.3 and 0.14 to 0.27 for the M10, H05 and I13 trials, respectively (Supplementary Fig. 2). The highest values were observed in M10 which experienced lower heat stress compared with H05 and I13. Unexpectedly, the lowest Stg values were found in H05 and not in I13, but the variability for this trait was reduced under intense heat stress in I13. The rate of senescence for the parents by environment is presented in Supplementary Fig. 3.

**Table 2:** Means and basic statistics for traits measured during the whole development of the Seri/Babax RILs, in five heat-stressed, irrigated environments

Trait	Parents means		RILs				Across environments	
	Seri	Bah ax	Mean^a^	Minimum^a^	Maximum^a^	*σ* ^a^	*h* ^2^	LSD
Staygreen	0.220	0.219	0.230	0.136	0.326	0.042	0.380	0.067
RS (NDVI/ °C day)	7.50 × 10^−4^	8.40 × 10^−4^	7.90 × 10^−4^	5.20 × 10^−4^	11.1 × 10^−4^	1.10 × 10^−4^	0.114	1.60 × 10^−4^
Gdecay (%)	31.3	30.5	31.2	18.0	44.0	5.27	0.284	7.94
Yield (g/m^2^)	235	258	238	159	317	32.5	0.773	33.0
KN (grain s/m^2^)	8325	7695	8185	4521	11807	1270	0.760	1238
TGW (g)	28.6	33.8	29.4	23.1	36.5	2.32	0.856	1.96
GFR(gm^−2^/day)	9.10	10.4	9.62	5.71	13.1	1.39	0.724	1.45
GFD (days)	28.4	27.8	27.5	24.1	31.3	1.23	0.430	1.48
NDVIv	0.516	0.621	0.603	0.514	0.667	0.030	0.740	0.031
NDVIg	0.417	0.435	0.432	0.347	0.533	0.034	0.738	0.029
Chlv (SPAD units)	43.9	43.5	43.4	38.8	47.0	1.57	0.316	2.30
Chlg (SPAD units)	46.0	47.2	46.6	40.6	51.6	2.12	0.453	3.12
CTv (°C)	26.3	26.2	26.5	25.2	27.7	0.483	0.575	0.515
CTg (°C)	31.3	31.2	31.3	30.0	32.9	0.519	0.546	0.665
Heading (dae)	52.7	52.5	52.7	46.9	59.6	2.52	0.938	1.60
Maturity (dae)	79.7	78.7	78.8	73.1	85.5	2.61	0.937	1.66
Height (cm)	61.2	69.1	66.0	56.2	75.9	3.92	0.824	3.54

*Stg* NDVI at physiological maturity, *RS* rate of senescence, *Gdecay* percentage of greenness lost at mid grainfilling, *KN* kernel number, *TGW* thousand grain weight, *GFR* grainfilling rate, *GFD* grainfilling duration, *NDVIv* normalized difference vegetative index during vegetative stage, *NDVIg* normalized difference vegetative index during grainfilling, *Chlv* chlorophyll content at vegetative stage (SPAD), *Chlg* chlorophyll content at grainfilling (SPAD), *CTv* canopy temperature at vegetative stage, *CTg* canopy temperature at grainfilling, *dae* days after emergence

^a^Values presented are the averages across each trial’s mean/minimum/maximum

**Table 3:** Phenotypic correlation (*r*_*P*_) for Stg and RS with performance traits by individual trial

	H05	M10	I13
Pearson’s correlation for the Stg			
Yield	0.275 (0.0003)	0.320 (<0.0001)	0.330 (<0.0001)
GFR	0.430 (<0.0001)	0.440 (<0.0001)	0.430 (<0.0001)
GFD	−0.350 (<0.0001)	−0.580 (<0.0001)	−0.400 (<0.0001)
KN	0.216 (0.0048)	0.130 (0.0906)	0.260 (0.0007)
TGW	0.160 (0.0415)	0.270 (0.0004)	0.056 ns
Pearson’s correlation for the RS			
Yield	0.488 (<0.0001)	0.243 (0.0014)	0.110 (0.154)
GFR	0.474 (<0.0001)	0.190 (0.013)	0.138 (0.073)
GFD	0.080 ns	0.005 ns	−0.130 (0.0906)
KN	0.429 (<0.0001)	0.026 ns	0.081 ns
TGW	0.123 (0.113)	0.240 (0.0014)	0.033 ns

The Pearson’s correlation for Stg (residual greenness at physiological maturity) and RS (rate of senescence) with yield, grainfilling rate (GFR), kernel number (KN) and kernel weight (TGW) are indicated for each of the three trials. In brackets the *p* values are shown. *ns* not significant. For RS the correlation was calculated using absolute values, i.e. positive correlations indicates larger trait values are associated with faster greenness decay

### Modelling NDVI across crop development

**Fig. 4:**
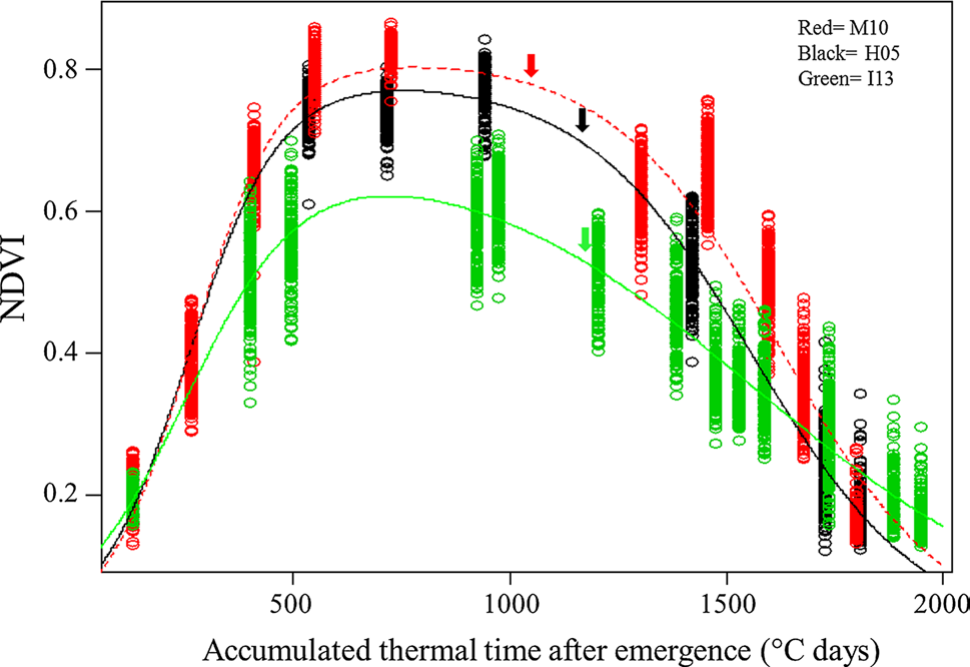
Modelling NDVI across the whole crop cycle. NDVI vs. thermal time (TT) was modelled for each of three trials of the Seri/Babax RILs population. Average days to heading for the environments are indicated by *arrows*

Individual measurements of NDVIv and NDVIg were plotted against thermal time and by regression analyses a single curve was fitted for the whole population for each environment. The performance of the NDVI trait across the cycle showed similar patterns in H05 and M10; major differences were observed in the NDVI pattern of the highest stressed environment, I13 (Fig. 4). Maximum NDVI was about 0.80 in M10 and 0.75 in H05, contrasting with I13 where the maximum NDVI was only 0.6. These maximum values were reached at about 750 degree-days in all environments.

**Fig. 5:**
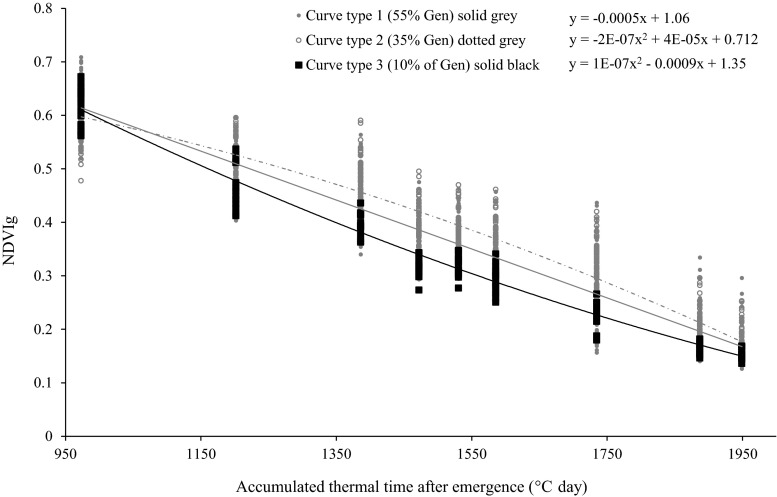
Greenness decay during grainfilling for Seri/Babax RILs in the I13 heat-stressed, irrigated environment. *Curves* represent the pattern for average NDVIg for RILs individually fitting one of the three different curve types. *Gen* genotypes

During grain filling, Seri showed lower initial NDVIg values than Babax at the same thermal time in the three environments (Supplementary Fig. 4). However, the decline in greenness in Seri was slower than the decline in Babax resulting in only marginally lower Stg for Seri. When modelling each mapping line separately, the 169 genotypes were observed to fit one of three types of curves best (Fig. 5). In the I13 environment, higher variation for type of curve was observed, given that the proportion of genotypes that fitted better to a linear curve (55 %) was close to the proportion of genotypes that fitted better to a parabolic curve (45 %). But when the heat stress was lower the diversity was reduced. In H05, 96 % of the population fitted a parabola best (curve type 2 and 3) and only 4 % fitted a linear model (curve type 1); while in M10 all the genotypes fitted a parabolic (curve type 2) curve best (data not shown).

To investigate relation between trait performance and NDVIg curve types, a subset of 53 genotypes with restricted range of phenology (average difference in heading date between groups was restricted to 1 day) was selected from the I13 environment in order to balance the number of genotypes included on each group. This environment was chosen because it exhibited a larger diversity for type of curve compared to M10 and H05. In an ANOVA, curve type was significantly related to yield (Table 4). Significant differences were found between genotype groups with different curve types, for yield, yield components and physiological traits (Table 4). The curve type with largest StgAUC, curve type 2, was associated with higher yield, KN, TGW, NDVIg, GFR and GFD. Significant differences were also detected for phenology and plant height, even though differences in heading time between groups were restricted.

**Table 4:** Comparative trait analysis across a subset of Seri/Babax RILs sorted by three different NDVIg curve types in the I13 heat-stressed, irrigated environment. Values in the table represent the average by type of curve best fit in the NDVIg—vs—dae regression analysis

Type of curve	*n*	StgAUC (NDVI × °C d)	Yield (g/m^2^)	Stg	RS (NDW°C d)	KN (grains/m^2^)	TGW (g)	NDVIg	Heading (days)	Maturity (days)	Height (cm)	GFR (g m^−2 ^/day)	GFD (days)
1	23	365 b	129 a	0.235 a	0.00048 a	4965 a	26.2 b	0.332 b	46 b	70 b	60 a	5.4 a	24.1 a
2	15	393 a	130 a	0.228 a	0.00051 a	4553 ab	28.7 a	0.362 a	47 a	72 a	61 a	5.2 ab	24.9 a
3	15	353 c	113 b	0.223 a	0.00043 b	4129 b	27.7 ab	0.313 c	46b	71 b	59 a	4.7 b	24.4 a
*p* value		<0.0001	0.0231	0.052	<0.0001	0.0095	0.0157	<0.0001	0.0079	<0.0001	0.128	0.0388	0.0808

*StgAUC* staygreen area under the curve with starting points at maximum NDVI, *Stg* NDVI at physiological maturity, *KN* kernel number, *TGW* thousand grain weight, *NDVIg* normalized difference vegetative index during grainfilling, *GFR* grainfilling rate, *GFD* grainfilling duration

### QTL mapping

The QTL mapping analysis was performed for 19 traits by single and by combined environments resulting in a total of 98 analyses (Trait × Environment combinations). A total of 193 QTL were identified with LOD > 2. Of these, 44 QTL were linked to Stg and staygreen associated traits, 37 QTL were associated with yield and yield components and the rest were related to other physiological parameters and phenology. Average LOD scores for all QTL associated with Stg and related traits, yield and yield components and with physiological traits were 3.5, 4.1 and 4.0, respectively. Across all QTL, for all traits and environments, the highest LOD score and the maximum phenotypic variance explained was 18.4 and 36.4 %, respectively, which was for a QTL on 1B for NDVIv. Additionally, 13 linkage groups contained two QTL located >30 cM apart for the same trait. A summary of results is presented as a matrix in Table 5. More detail about QTL with LOD > 3.5 is presented on Table 6; this table shows the related marker(s), maximum variances, size of effects as well as the increasing allele for each QTL. Except for H05, the maximum variances explained in all environments were found for QTL related to traits other than yield.

**Table 5:** Co-location of QTL for mapped traits

Chromosome Total Env (***n*** **)**	Stg 3	RS 3	TotalAUC 3	StgAUC 3	Gdecay 3	Yield 5	KN 5	TGW 5	GFR 5	GFD 5	NDVIv 5	NDVIg 5	Chlv 3	Chlg 3	CTv 5	CTg 4	Heading 5	Maturity 5	Height 5
1A			**1** **+** **C**	**1** **+** **C**			2	**5** **+** **C**		1	1	1		1 + C			C	1	3 + C
1B		**1** **+** **C**	**1** **+** **C**		**2** **+** **C**	**3** **+** **C**	**4** **+** **C**	**C**	**4** **+** **C**	1 + C	**5** **+** **C**	1		**3** **+** **C**	**1** **+** **C**		1 + C	2 + C	
1D	1 + C		C			1			2	1	**1**		1		**3** **+** **C**		**1** **+** **C**	1 + C	
2A	1	**1** **+** **C**	**1** **+** **C**	**1** **+** **C**		C	1 + C	1				**3**		1					**1**
2B	**C**	**2**	C	**1** **+** **C**	**1** **+** **C**	1	1	**3** **+** **C**	**1**	2 + C	1	**2** **+** **C**	1 + C	**2**	**2** **+** **C**	1	**2** **+** **C**	**4** **+** **C**	**2**
2D	1 + C	**1** **+** **C**			**2** **+** **C**	1	C	**2**	1	**1**	**2** **+** **C**		**2** **+** **C**						
3A			1									**2**				1			**3** **+** **C**
3B		1			1 + C	**2** **+** **C**	**1** **+** **C**	**5** **+** **C**	**1** **+** **C**		**2** **+** **C**	1 + C	**1** **+** **C**	**1** **+** **C**	**2** **+** **C**		1		**2** **+** **C**
4A		**C**	1	1	**1** **+** **C**	**3** **+** **C**	**3** **+** **C**	**2** **+** **C**	**3** **+** **C**		**2** **+** **C**	**1** **+** **C**		1	**4** **+** **C**	**2** **+** **C**	**1** **+** **C**	1	
4B	**2** **+** **C**		C	C	1	1	**2** **+** **C**	**3** **+** **C**		**1**	**1** **+** **C**	**1**	**2**					1	**3** **+** **C**
4D	1											**1**					**4**	2	
5A	1				1 + C	**1** **+** **C**			**2** **+** **C**	1		1			1	2	1 + C	**2** **+** **C**	2 + C
5B			**2** **+** **C**	**2** **+** **C**		1		**1** **+** **C**	1		**2** **+** **C**	2	**1** **+** **C**						**4** **+** **C**
5D						1				1						**2** **+** **C**	**5** **+** **C**	**3** **+** **C**	1
6A	1		1 + C					1		**2** **+** **C**	**2** **+** **C**	1	**2** **+** **C**		1		**3** **+** **C**	2	1
6B		2			1	**4** **+** **C**	**3** **+** **C**	1	**2** **+** **C**	1		**C**							
6D							**3** **+** **C**	**2** **+** **C**	1	**2** **+** **C**									
7A			**1** **+** **C**	1		**1**		**1** **+** **C**			**3** **+** **C**	1 + C	1	1		1			2
7B																			
7D	**3** **+** **C**			1	**C**			**3** **+** **C**		**2** **+** **C**	**1**	**4** **+** **C**				**1** **+** **C**	**5** **+** **C**	**5** **+** **C**	**2** **+** **C**
Max% var explained	15.0	10.4	11.3	10.5	11.1	16.8	18.0	11.9	16.2	9.6	36.4	11.7	11.8	13.5	16.8	14.6	18.1	13.9	7.8
Associated marker	acc/cat-10	act/ctc7	wPt-0103	gwm526	agg/cat-4	acc/cat4	agc/cta-9	aca/cta-2	aag/ctc6	wPt-0298	gwm131	acc/cat-10	gwm617b	barc0164	aac/ctg-3	acc/cat10	acc/cat10	acc/cat10	aca/caa-6
Chromosome	7D	1B	5B	2A	1B	1B	1B	1A	1B	2D	1B	7D	6A	3B	4A	7D	7D	7D	3A

The number of environments where a QTL was identified is written, followed by +C if the QTL was also detected in the combined QTL analysis across environments

Bold values: chromosomes where a consistent QTL (LOD > 3.5 in at least one environment) was detected. The number of environments where a given trait was recorded is indicated in the first row below trait names; in the table the number of environments where a QTL was identified is written followed by +C if the QTL was also detected in the combined QTL analysis across environment

*Stg* staygreen at physiological maturity, *RS* rate of senescence, *TotalAUC* total area under the curve with starting points at crop establishment, *StgAUC* area under the NDVIg curve with starting points at maximum NDVIg, *Gdecay* percentage of greenness lost at mid grainfilling, *KN* kernel number, *TGW* thousand grain weight, *GFR* grainfilling rate, *GFD* grainfilling duration, *NDVIv* normalized difference vegetative index during vegetative stage, *NDVIg* normalized difference vegetative index during grainfilling, *Chlv* chlorophyll content at vegetative stage (SPAD), *Chlg* chlorophyll content at grainfilling (SPAD), *CTv* canopy temperature at vegetative stage, *CTg* canopy temperature at grainfilling

**Table 6:** QTL with LOD > 3.5 identified for all traits in the Seri/Babax population grown under M10, H05, H11, I06 and I13 heat-stressed, irrigated environments

QTL location	Linked markers with LOD>3.5	Position (cM)	Max % of variance explained				QTL location	Linked markers with LOD>3.5	Position (cM)	Max % of variance explained			
			R^2^	Effect	Allele	Env				R^2^	Effect	Allele	Env
Stg							GFD (days)						
4B	aac/ctc-9	12.8	10.6	0.021	Babax	H05	2D	wPt-0298	70.8	9.65	0.182	Seri	M10
7D	acc/cat-10	2.73	15.0	0.025	Seri	H05	4B	agc/cag-2	12.0	7.70	0.336	Seti	H11
RS (NDVI/°C d)							6D	cfd0188	41.4	9.60	0.464	Seri	106
1B	act/ctc-7	61.1	10.4	0.000	Babax	I13	7D	acc/cat-10	2.73	8.50	0.366	Babax	I13
	aag/ctg-14	61.2					NDVIv						
2A	gwm526	1.34	3.50	0.000	Seri	H05	1B	gwm131	64.2	36.4	0.024	Babax	H11
2B	aag/ctg-12	37.9	8.50	0.000	Babax	M10		aag/ctc-6	61.8				
	wPt-7750	27.0						agg/cat-4	62.2				
2D	wPt-2644	74.6	6.10	0.000	Seri	H05	1D	wPt-1770	9.08	6.70	0.009	Seri	I13
TotalAUC (NDVI × °C d)							3B^a^	wPt-8021	40.2	6.60	0.008	Babax	I06
1A	wPt-0432	120	6.90	11.6	Seri	M10	4A^a^	agg/cta-12	13.6	20.1	0.013	Babax	I06
1B	aca/cta-9	59.7	5.90	10.4	Babax	H05	4B	gwm006a	23.8	6.20	0.009	Babax	I13
2A	gwm526 (1.34)	1.34	6.80	16.9	Babax	I13	5B	aag/ctg-11	6.88	14.1	0.014	Babax	I13
5B	wPt-0103 (10.92)	10.9	11.3	21.7	Babax	I13	6A	wPt-7599	50.8	6.40	0.009	Seri	I13
7A	barc121 (97.45)	97.5	9.20	19.6	Seri	113	7A^a^	aag/cta-2	98.8	6.90	0.009	Seri	I13
StgAUC (NDVI × °C d)							7D	acc/ctc-7	11.7	7.90	0.005	Seri	M10
1A	wPt-8644	115	6.60	10.0	Seri	M10	NDVIg						
2A	gwm526	1.34	10.5	11.9	Babax	I13	2A	gwm526	1.34	10.1	0.013	Babax	I13
4A	act/cag-3	13.2	7.70	9.5	Babax	H05	2B	wPt-5680	40.9	8.10	0.011	Seri	I13
5B	wPt-0103	10.9	7.30	9.9	Babax	I13		aag/ctg-12	37.9				
Gdecay (%)							3A	wPt-7341	2.42	5.20	0.008	Babax	H11
1B	agg/cat-4	62.2	11.1	1.29	Babax	M10	4B	aac/ctc-9	12.8	5.90	0.008	Seri	M10
2B^a^	wPt-7750	27.0	9.80	1.89	Babax	I13	4D	gdm0129	0.870	7.50	0.009	Babax	H11
2D^a^	gwm102	59.6	6.30	1.51	Seri	I13	7D	acc/cat-10	2.73	11.7	0.011	Babax	I06
4A	act/cag-3	13.2	8.80	1.74	Seri	H05	Chlv (Spad units)						
4B	gwm006a	23.8	7.30	1.63	Babax	I13	2D	wPt-2644	74.6	10.8	0.477	Seri	H05
6B	aac/ctc-3	83.0	8.20	1.73	Seri	I13	3B	wPt-1940	112	7.00	0.467	Babax	I06
Yield (g/m^2^)							4B^a^	aag/cta-5	11.6	9.40	0.445	Babax	H05
1B	acc/cat-4	61.7	16.8	16.6	Babax	H05	5B	wPt-0103	10.9	8.90	0.435	Seri	H05
	acg/cta-2	61.5					6A^a^	gwm617b	28.4	11.8	0.503	Seri	I13
	agg/cac-3	65.4					Chlg (Spad units						
3B^a^	gwm301e	44.6	8.90	12.4	Babax	I06	1B	acc/cat-4	61.7	12.6	0.580	Seri	H05
4A^a^	wmc048d	12.9	16.1	16.7	Babax	I06		wPt-7529	61.2				
	aca/cac-6	103					2B	acg/cta-1	35.7	9.50	0.546	Babax	I06
4B	wmc048a	9.70	7.00	10.2	Seri	H11	3B	barc0164	I13	13.5	0.601	Babax	H05
6B^a^	wPt-2786	36.4	7.30	10.4	Seri	H11	CTv (°C)						
	aac/ctc-3	83.0					1B	acc/cat-4	61.7	16.6	0.157	Seri	H05
	barc0178	90.3					1D^a^	wPt-9380	41.8	11.2	0.154	Babax	H11
7A	aag/cta-3	115	8.50	11.3	Seri	H11	2B	acc/ctg-4	25.2	5.90	0.120	Babax	I06
KN (grains/m^2^)							3B	gwm301e	44.6	9.90	0.156	Seri	I06
1B^a^	agc/cta-9	66.4	18.0	601	Babax	H11	4A	aac/ctg-3	12.9	16.8	0.202	Seri	I06
	gwm301b	61.9						wmc048d	13.8				
3B^a^	barc147	87.7	7.60	449	Seri	I06	CTg(°C)						
4A^a^	wmc048d	12.9	12.7	582	Babax	I06	4A^a^	wmc048d	12.9	9.70	0.123	Seri	M10
4B	gwm375	14.1	7.10	316	Babax	M10		agg/cta-12	13.6				
6B	barc0178	90.3	8.50	380	Seri	H05	5D	wPt-1400	13.0	7.20	0.220	Seri	H05
	agg/cat-8	64.5					7D	acc/cat-10	2.73	14.6	0.314	Seri	H05
TGW (g)							Heading (dae)						
1A	aca/cta-2	31.2	11.9	0.897	Babax	I06	2B	wPt-7750	27.0	8.00	0.576	Seri	H11
	agg/cac-6	42.3					4D	cfd023	2.25	9.10	0.725	Seri	I13
	aca/cag-13	53.8					5D	wPt-5505	12.6	8.60	0.704	Babax	I13
2B^a^	acc/ctc-2	24.2	6.70	0.531	Babax	M10		wPt-1400	13.0				
2D	gwm102	59.6	5.00	0.457	Seri	M10	6A	wPt-0696	34.2	9.80	0.751	Babax	I13
3B^a^	agg/cat-3	89.9	7.40	0.632	Babax	H05	7D	acc/cat-10	2.73	18.1	0.824	Babax	M10
	wPt-2757	86.8						acc/ctc-7	11.7				
4A	act/cag-1	75.7	5.40	0.606	Babax	I06							
4B	aag/cta-5	11.6	10.8	0.756	Seri	I13							
	wPt-1708	9.27											
TGW (g)							Maturity (dae)						
5B	gwm371	14.5	6.60	0.592	Babax	I13	2B	gwm388	34.2	9.30	0.742	Seri	H11
6B	barc0178	90.3	10.9	0.767	Babax	H05	5D	wPt-5505	12.6	8.10	0.690	Babax	H11
6D	cfd0188	41.4	8.80	0.684	Babax	I13		wPt-1400	13.0				
	gwm325	37.0					7D	acc/cat-10	2.73	13.9	1.22	Babax	H05
7A	aca/cag-10	78.6	6.20	0.581	Seri	I13	Height (cm)						
7D	cfd0014	41.8	9.00	0.699	Seri	H05	2A	gwm526	1.34	7.00	1.01	Babax	H11
GFR (g m^−2^/Vday)							2B	aca/ctg-1	37.5	7.30	0.989	Babax	M10
1B	aag/ctc-6	61.8	16.2	0.763	Babax	H05	3A	aca/caa-6	0.350	7.80	1.33	Seri	I06
	gwm131	64.2					4B	aag/cta-5	11.6	7.50	0.787	Seri	I13
	agc/cta-9	66.4					5B	gwm274	5.51	7.50	1.23	Babax	H05
2B	agg/cac-5	28.6	7.00	0.296	Babax	M10		wPt-9814	0.190				
3B^a^	gwm301e	44.6	7.20	0.470	Babax	I06		gwm133	7.47				
	gwm389	92.9	5.50	0.411	Seri	I06	7D	acc/cat-10	2.73	7.00	1.18	Babax	H05
4A^a^	wmc048d	12.9	15.1	0.682	Babax	I06							
	gha44	15.2											
6B	wPt-8412	61.2	6.60	0.486	Seri	H05							
	agc/cta-4	79.0											

For each QTL all linked markers with LOD > 3.5 are listed. Only the environment where the maximum variance explained was detected for a given QTL is indicated together with its corresponding effect and allele contributing to increase the trait. For QTL with more than one listed marker the first is the marker related to the maximum *R* ^2^

*Stg* staygreen at physiological maturity, *RS* rate of senescence, *TotalAUC* total area under the curve with starting points at crop establishment, *StgAUC* staygreen area under the curve with starting points at maximum NDVI, *Gdecay* percentage of greenness lost at mid grainfilling, *KN* kernel number, *TGW* thousand grain weight, *GFR* grainfilling rate, *GFD* grainfilling duration, *NDVIv* normalized difference vegetative index during vegetative stage, *NDVIg* normalized difference vegetative index during grainfilling, *Chlv* chlorophyll content at vegetative stage (SPAD), *Chlg* chlorophyll content at grainfilling (SPAD), *CTv* canopy temperature at vegetative stage, *CTg* canopy temperature at grainfilling, *dae* days after emergence

^a^Chromosomes with two QTL for the same trait since distances between associated markers was >30 cM

### QTL for staygreen traits

QTL for Stg were located on chromosomes 2A, 4B, 4D, 6A and 7D. The largest phenotypic variance (15 %) was for a locus on 7D. This was also the most repeatable Stg QTL detected (two of three environments plus the combined analysis). Stg related traits such as RS, StgAUC, TotalAUC and Gdecay gave 9, 8, 11 and 11 QTL, respectively. The 4B and 7D loci seemed to be the main genomic regions controlling Stg related traits, given that those QTL were identified for multiple environments and traits (Table 5). A QTL on 1B explained around 10 % of the phenotypic variance for both RS and Gdecay. On 2B a QTL was detected for RS, TotalAUC, StgAUC and also for Gdecay where the greatest variance explained was about 10 % (for Gdecay). Most of the QTL for StgAUC and TotalAUC had LOD values greater than 3.5. QTL on 5B explained 11.3 % of the variance for TotalAUC and 7.3 % of variance for StgAUC (Table 6). For StgAUC the maximum phenotypic variance, 10.5 %, was explained by a QTL on 2A (Table 5). Considering all the environments, alleles from both parents contributed equally to Stg across the genome (Table 6).

### QTL for agronomic and physiological traits and co-location with QTL for staygreen traits

**Fig. 6:**
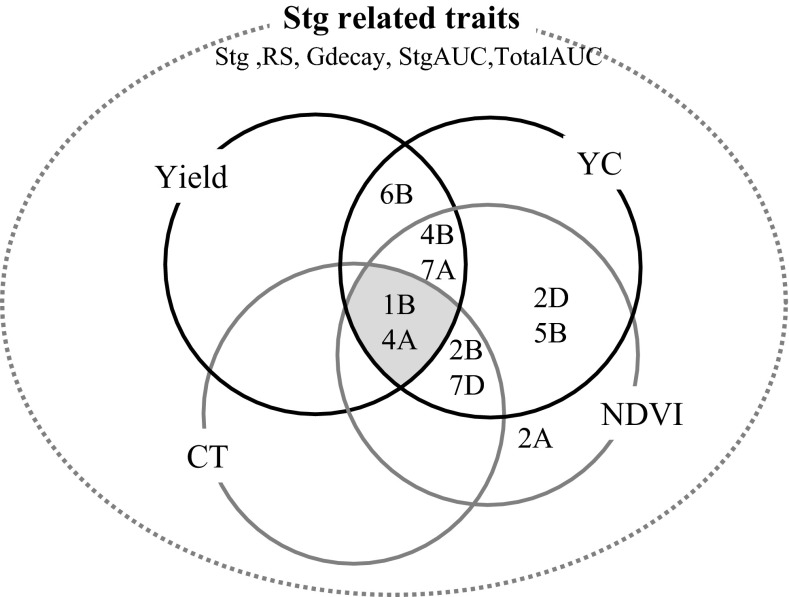
Genetic overlap between QTL loci controlling staygreen and related traits with those controlling other traits. Only consistent QTL (LOD > 3.5) are represented, and co-location is defined as positions <30 cM. *YC* yield components (kernel number and grain weight), *CT* canopy temperature (CTv and CTg), *NDVI* normalized difference vegetation index (NDVIv and NDVIg). Staygreen related traits included: Stg, RS, Gdecay, StgAUC and TotalAUC are listed at the *top*

A number of QTL associated with agronomic and physiological traits were found co-located (linked to markers <30 cM) with QTL for Stg and staygreen related traits (Table 5). Figure 6 shows a Venn diagram summarizing these genetic overlaps. The 1B, 3B, 4A, 4B and 6B genomic regions appeared to be the most important ones controlling yield and yield components based on repeatability and significance (Table 5). Yield QTL co-located with QTL for Stg and staygreen related traits on 1B, 2A, 2B, 3B, 4A, 4B, 5A, 5B, 6B and 7A, and the QTL on 1B, explained the greatest variances for yield (linked to markers at 61.71–65.36 cM), KN (60.73–66.35 cM) and GFR (61.81–66.35 cM) (Table 6). This yield QTL on 1B appeared in three of five environments plus in the combined analysis, and was also found at or near QTL for RS, TotalAUC and Gdecay. The strongest effects for yield (16.5 g/m^2^) were found on 1B and 4A. For TGW, a QTL on 1A explained close to 12 % of variance and had an additive effect of almost 1 g in the M10 and I06 environments. QTL for StgAUC and TotalAUC were also found on chromosome 1A but >30 cM distant from the QTL for TGW. In total, 28 QTL were identified for NDVI, 12 for NDVIv and 16 for NDVIg; most of these QTL showed LOD > 3.5. On 1B, a major QTL for early ground cover, defined by NDVIv, was found in the same region as QTL for RS, TotalAUC and Gdecay; for all the traits the QTL were linked to markers found between 59.7 and 64.2 cM (Table 6), indicating co-location. This NDVIv QTL on 1B explained more than 36 % of phenotypic variance for the trait. On the other hand the maximum variance for NDVIg (12 %) was explained by a QTL on 7D (linked to one marker on 2.73 cM) which co-located with QTL for Stg, RS and Gdecay (linked to markers at 2.73–11.1 cM).

On chromosomes 1B, 2B and 3B, there was co-location of chlorophyll content QTL (LOD > 3.5), defined by Chlv and Chlg, with Stg QTL related traits; in these three regions the Sgt and Chl QTL were associated with closely linked markers (at ~60, 40 and 113 cM for 1B, 2B and 3B, respectively). Almost 12 % of variance for Chlv was explained by a QTL on 6A, while a QTL on 3B explained about 14 % of the variance for Chlg. Eight QTL were detected for CTv and eight for CTg. Average LOD scores for all QTL related to canopy temperature was 4.2. For CTg the maximum variance was 15 %, explained by a QTL on 7D (at 2.73 cM), which was co-located with a number of QTL for Stg and related traits (at 2.73–11.1 cM). Additionally, the 4A region showed two regions affecting both CTg and yield, the first being located close to 13 cM and the other at around the 111 cM. The maximum variance explained for CTv was for loci on 1B and 4A, each explaining 17 % of the variance. Opposite to the 1B QTL, the QTL on 4A was repeatedly detected for CTv and CTg and in all environments, excepted in H11. The two CTv QTL on 1B and 4A co-located with QTL for yield showing the strongest effects for the trait, but did not co-locate with Stg QTL. The QTL detected for CTv at ~61 cM on 1B also controlled RS. The QTL for CTv and CTg on chromosome 4A co-located with QTL for RS, StgAUC and TotalAUC; only the QTL for RS seems to be different, given the large distances between QTL; The CTv and CTg QTL were found at 13–15 cM while the QTL for RS was located at 72 cM. QTL for Gdecay coincided with QTL for CTv and CTg on 1B, 2B, 3B, 4A and 7D, and in all cases the linked markers were closely located, indicating that it was the same QTL. Plant height was mainly controlled by loci on 3A, 4B and 5B. The strongest QTL for plant height was found on 3A, explained about 8 % of phenotypic variance for the trait and had an additive effect of 1.3 cm. This QTL on 3A was not co-located with any QTL for Stg or related traits of LOD > 3.5, or for yield or yield components. However the height QTL on 4B and 5B co-located with QTL for Stg, TotalAUC, StgAUC, Gdecay, yield, TGW and KN.

### QTL for plant phenology

Plant phenology QTL (date of heading and maturity) were positioned across the Seri/Babax genome but with small individual effects (<1.5 days, see Table 6). A QTL on 7D explained the highest variances for both heading and maturity. Based on repeatability and significance it seems that plant phenology was mainly controlled by the 2B, 5D and 7D genomic regions. The consistent QTL (LOD > 3.5) on 2B, 4A and 7D co-located with consistent QTL for Stg and all related traits. QTL for all these traits were found linked to markers at 26.8–40.9 cM on 2B, at 12.92–23.65 cM on 4A and on 2.73–11.7 cM on 7D. The phenology QTL on 5D did not co-locate with any QTL for Stg or related traits.

### Allele significance for all traits

Considering all the environments, alleles from both parents contributed equally to Stg across the genome (Table 6). QTL for Stg, StgAUC and TotalAUC mostly had Babax contributing the increasing allele i.e., these alleles favoured higher areas under the NDVI curve during the whole crop cycle (TotalAUC) and also during the greenness decay phase (StgAUC). Regarding yield, TGW and KN these traits were increased by alleles from both parents across the genome; however, Babax alleles tended to contribute the highest positive effects at loci explaining the maximum variances. Similarly, both parents contributed to increases in NDVI during both the vegetative and the grainfilling stages, depending on the locus. On the other hand, increases in canopy temperature were largely contributed by Seri alleles.

## Discussion

### Understanding the staygreen mechanism in the Seri/Babax population—association with yield and plant performance

The staygreen phenotype has been associated with improved performance of several species under heat stress (Reynolds et al. 2000; Kumari et al. 2013) and in the current study there was a positive and significant association of Stg with yield and yield components (Table 3). However in order to properly exploit the potential of the staygreen trait, a clearer understanding of the underlying mechanisms for the staygreen phenotype in the context of the cumulative effect of traits contributing to yield maintenance in stressed environments is needed. The current study found Stg to be positively associated with high yield, TGW, GFD, KN, and low CT. While heat stress conditions can reduce the grain number due to seed abortion or reduced grain set (Hays et al. 2007; Tashiro and Wardlaw 1990) crop productivity is also related to longer grainfilling periods and faster grainfilling rates, so it is expected that under heat stress, staygreen traits and green tissue area contribute to heavier grains (Kumari et al. 2013). Canopy temperature depression has also been found to be positively and strongly correlated with staygreen traits suggesting a possible link with root development patterns in bread wheat (Christopher et al. 2008; Kumari et al. 2013), as found in sorghum staygreen genotypes (Borrell et al. 2014a). Herein, the canopy temperature during the vegetative stage (CTv) was also found to be associated with RS and with NDVIv (Supplementary Table 1) further supporting the hypothesis that the RS staygreen attribute in wheat is primarily a consequence of the initial amount of greenness (total biomass and chlorophyll) potentially available for filling the grains. This was supported by the fact that genotypes with cooler CTv tended to have higher initial greenness and biomass (NDVIv) and faster rates of senescence during grain filling.

NDVI is an integrative measure of chlorophyll and total plant biomass, confirmed by a significant positive correlation between NDVIg and Chlg and height (Supplementary Table 1). The absolute rate of senescence (RS) was positively correlated with yield in the Seri/Babax population (Table 3), showing that genotypes with higher yields tended to lose chlorophyll faster. Higher absolute RS was also observed in genotypes with higher NDVIv, StgAUC and TotalAUC (Supplementary Table 1) showing that despite higher rates of NDVI decay during grain filling in these genotypes, the total amount of initial NDVIg was higher allowing for higher amounts of photosynthesis per unit degree day to fill grains. Interestingly, higher RS did not result in a faster arrival to maturity (associations of RS with days to maturity were not significant). This suggests that among the Seri/Babax progeny, genotypes with a staygreen phenotype were characterized by a high initial greenness, high StgAUC and TotalAUC and high RS, while attaining maturity within a similar timeframe, compared to non staygreen genotypes. In most species studied so far, a very conservative response has been observed for the staygreen phenotype with low RS and delayed onset of senescence (Thomas and Ougham 2014). However, the wheat Seri/Babax population grown in warm and irrigated environments showed a pattern of staygreen where higher initial greenness is lost at a higher rate without really accelerating time to maturity (Supplementary Table 1, NDVIv and Maturity, *r*_*P*_ = 0.13, *p* = 0.089). Nonetheless, analysis across all environments showed low heritability for Stg especially for RS, similarly to results reported by Lopes and Reynolds (2012) in one staygreen study performed in the same population. Moderate and high heritability was found for physiological and agronomic traits (Table 2).

### Differentiating patterns of plant greenness decay

Interpretation of staygreen would be most straightforward when dynamic traits fit a linear model. However during the grainfilling phase plant greenness decay patterns sometimes fitted non-linear models best. Non-linear regression curves have been previously used to describe the percent of greenness retained during grainfilling (Vijayalakshmi et al. 2010). Additionally, a number of genotypes from the Seri/Babax population were found to fit best a parabolic model in the M10, H05 and I13 environments. Parabolic curves were observed in two of the 3 years in which the Stg attribute was analyzed. Interestingly, the tendency to follow a particular pattern was related to heat stress intensity. Furthermore, the same genotype could fit different curves, depending of the environment, suggesting high *G* × *E* for staygreen traits, as reported in previous studies (Bogard et al. 2011; Kumar et al. 2010). According to our modelling results the best time to screen staygreen parameters under heat-stressed, irrigated environments is around mid grainfilling (1200–1550 dae), given that in this period was observed highest resolution in the greenness canopy dynamics between genotypes (Fig. 4, 5). The latter was supported by co-location of QTL for yield and performance traits with QTL for Gdecay; this parameter estimates the percentage of greenness lost (from the maximum) at mid grainfilling and Table 5 showed that the main region controlling Gdecay, RS, yield, KN and GFR was 1B; several additional regions of minor effect were also found in common between these traits. Notwithstanding, for a completer understanding of the canopy dynamics it is suggested to start NDVI recordings when the maximum is reached (in these study it was around the about 750 degree-days) and extend the measurements after physiological maturity. The largest genetic diversity for type of curve was observed in the I13 environment which experienced the highest temperatures; in this environment linear and non-linear models applied to an almost equal proportion of genotypes. Lower diversity for the type of curve was observed in the H05 environment in which heat stress was moderate and in which only 4 % of the population fitted a linear model (curve type 1). In M10, which was the least heat stressed environment, the whole population fitted a non-linear model best (data not shown).

A curve type 2 (see Fig. 5) during the decay phase resulted in larger area under the greenness curve (StgAUC) which would have allowed more photosynthesis, thus explaining the association of this curve type with higher grain yields (Table 4) (Kumari et al. 2013). By contrast, the lower StgAUC observed for curve type 3 resulted in lower photosynthetic area and genotypes with reduced grain number (KN) (Table 4). The classification of staygreen into four functional types is highly descriptive but in reality it is quite hard to classify a genotype into one or another group because the staygreen phenotype often results from a combination of two or more types (Thomas and Howarth 2000). Additionally, it is important to take into account that the Stg and RS traits by themselves cannot completely describe the staygreen attribute given the high relevance of the initial greenness value, as observed in the current study.

### Genetic basis of plant greenness decay: QTL mapping

Heat tolerance is a complex trait influenced by different component traits. Increasing temperatures accelerate plant development and decrease the length and amount of green biomass (through decreased organ size and plant height). The main chromosome regions controlling staygreen related traits in this wheat population were generally co-located with regions controlling agronomic and physiological attributes. Different staygreen traits were calculated and QTL mapped, including the residual greenness at maturity (Stg), the rate of senescence (RS), the green area under the curve (StgAUC) and the percentage of greenness lost at mid grainfilling (Gdecay)—all estimated from NDVI decay curves. The maximum phenotypic variance for any staygreen related QTL was detected on chromosome 7D associated with Stg; this locus has been previously described as associated with permanence of greenness under high temperatures (Vijayalakshmi et al. 2010; Kumar et al. 2010). In the current study, this Stg QTL on 7D co-located with a QTL for NDVIg, CTg (Table 5) and days to heading. Kumari et al. (2013) reported that staygreen in bread wheat was associated with high canopy temperature depression (CTD) such that the warmer plants tended to be non staygreen. There is evidence in sorghum that staygreen genes overlap with root architecture genes (Mace et al. 2012), for example, QTL for root nodal angle have been found to be co-located with Stg QTL including the Stg4 QTL associated with biomass partitioning between root and shoot (Borrell et al. 2014b). In the present study, the 7D region also controlled Gdecay and StgAUC as well as CTg, with the Seri allele being positive. Gdecay and CTg were positively correlated in the Seri/Babax population indicating that cooler genotypes tended to lose a smaller percentage of greenness in the first half of the grainfilling period. Gdecay and CTg controlled by the QTL on 7D seemed to be affected by plant phenology (Lopes et al. 2013) given the co-location of a main QTL for heading and maturity here (Table 5), but there was no effect of phenology in the 4A region where a consistent QTL was identified for Gdecay and CTg.

The highest phenotypic variability explained for Gdecay (11.1 %) and RS (10.6 %) was detected on the 1B chromosome. Chromosome 1B has been reported to control a number of performance traits. Yang et al. (2002) found a QTL for grain filling duration on the short arm of chromosomes 1B which co-located with a number of QTL for Stg related traits from this study. Moreover, this QTL on chromosome 1B was co-located with yield, Chlg, NDVIv, CTv, Gdecay and KN. The 1B region also has been associated with SPAD chlorophyll content (Talukder et al. 2014) and Pinto et al. (2010) reported several QTL on 1B for canopy temperature, yield, and chlorophyll content at the grain filling stage in the Seri/Babax population. Common QTL for Stg related traits, yield, yield components and physiological characters indicate a common genetic basis for these attributes. The strongest QTL for yield detected in the current study was found on chromosome 1B and interestingly, it co-located with a QTL for green leaf duration detected in a previous study of spring wheat grown under heat stress in greenhouse experiments (Naruoka et al. 2012). The calculation and mapping of diverse staygreen associated parameters across the crop cycle allowed to determine if these parameters are under independent genetic controls in the Seri/Babax population. Our study showed that the strongest regions controlling StAUC and TotalAUC are different from those with largest effects for Stg, Gdecay and RS which suggest independent genetic controls for these traits. However, co-location of QTL for these parameters were also identified across the wheat genome which indicate minor overlapping of genes. In conjunction it seems that the mapping of diverse parameters associated to the staygreen attribute contribute with additional and valuable information that could be lost if the investigation is limited to the staygreen (Stg) study per se. For example, the 1B region was found to contain main genetic controls for yield and other agronomic traits and QTL for RS, TotalAUC and StgAUC were identified on 1B but not for Stg (Table 5).

In agreement with our results (Table 5), Naruoka et al. (2012) found that the 4A and 3B chromosomes controlled green leaf duration in spring wheat grown under heat and also drought stress; in the Seri/Babax population the 4A and 3B chromosomes seemed to contain genes driving StgAUC, RS and Gdecay. These two genomic regions also showed QTL for yield, yield components, NDVI, GFR, chlorophyll content and canopy temperature which coincided with results from Pinto et al. (2010). During leaf senescence the mechanisms that protect the chlorophyll molecule from photodamage fail and result in leaf yellowing (Thomas and Howarth 2000). In some species, the staygreen phenotype can be conferred by genetic deletions of the locus encoding phaeophorbide *a* oxygenase (PaO), the main regulatory enzyme for chlorophyll catabolism (Vicentini et al. 1995; Roca et al. 2004; Thomas and Howarth 2000). However, the genetic basis of the staygreen phenotype is complex and differs from one species to another. Multiple staygreen genes (SGR) have been identified in several species, but the number of staygreen genes varies between species and homologos genes do not always result in increased greenness persistence. This may be because staygreen genes may also have different functions from one species to another; an example of this is in *Arabidopsis* where over-expression of the SGR2 gene results in a staygreen phenotype whereas over-expression of the SGR1 gene promotes leaf yellowing (Sakuraba et al. 2015). The physiological and biochemical mechanisms by which the staygreen genes affect chlorophyll degradation are unclear but various studies seem to indicate the involvement of a multi-protein complex containing chlorophyll catabolic enzymes (CCEs), the product of the staygreen gene 1 (SGR1) and light-harvesting complex subunits of photosystem II (LHCII). Apparently, this complex channels phototoxic Chl intermediates during chlorophyll catabolism (Sakuraba et al. 2012).

### Cosmetic and functional staygreen

Studies have shown that the staygreen phenotype includes a genetic component affected by the phenological clock of the plant and a second component un-related to plant developmental stage. In the current study consistent QTL for staygreen related traits on 2A, 2D, 5B, 6B and 7A were not co-located with phenology QTL; while consistent QTL for staygreen related traits and consistent QTL for heading and maturity co-located on 2B, 4A, 4D and 7D. In general terms, earliness in the Seri/Babax population was associated with longer GFD. Overlapping genomic regions for plant phenology and staygreen attributes suggest common genes controlling these traits. In *Festuca pratensis,* staygreen independent from phenology has been reported as a recessive character generated by changes in a gene regulating the pathway of chlorophyll degradation (Vicentini et al. 1995); *Lolium* and *Festuca* staygreen mutants show expression of the PaO enzyme but with reduced activity (Vicentini et al. 1995; Roca et al. 2004). However, the underlying mechanism associated with the staygreen character seems to vary (Thomas and Howarth 2000). In soybean for example, staygreen can be the result of a cytoplasmic mutation, *CytG,* which makes the chlorophyll *b* structure more stable (Guiamét et al. 1991). The staygreen of these mutants may be classified as Type C or *cosmetic staygreen* (Sakuraba et al. 2015; Thomas and Howarth 2000) which is characterized by the permanence of the greenness, but with unaffected loss of photosynthetic function. Mutant lines have also been used to study staygreen in rice (Cha et al. 2002), wheat (Spano et al. 2003; Thomas et al. 2002; Rampino et al. 2006; Tian et al. 2012), *Arabidopsis* (Grbic and Bleecker 1995) and *Festuca* (Hauck et al. 1997). However, if the genetic lesion resulting in plant greenness persistence is also associated with improved plant performance, the staygreen is classified as *functional staygreen.* An example of functional staygreen is in sorghum where some genotypes remain green and give higher grain weights than the non staygreen genotypes (Duncan et al. 1981; Borrell et al. 2000). In the Seri/Babax population functional staygreen may be controlled by chromosomes where common QTL for Stg, yield and yield components were detected, such as 4B. On the contrary, the staygreen phenotype was unlinked to yield improvement on chromosome 7D suggesting that the locus controlled the cosmetic persistence of greenness.

The staygreen character is a complex trait; its expression is environment dependent suggesting high G × E interaction (Christopher et al. 2008; Bogard et al. 2011). For example, in sorghum the staygreen attribute is only observed under drought conditions (van Oosterom et al. 1996). In the current study, it was observed that the greenness decay pattern of particular genotypes varied with the growth conditions, resulting in different types of fitted curves (Fig. 5) when grown under moderate, hot or intense heat stress.

## Conclusions

Results from this study showed the staygreen attribute to be positively and significantly associated with yield and yield components in bread wheat grown under heat-stressed, irrigated conditions. The NDVI decay trend during grainfilling showed genotypic differences within the Seri/Babax population, and that the type of curve followed during greenness decay was strongly associated with general plant performance parameters. However, the type-curve for greenness decay is highly environment dependent. The association of the Stg character, the rate of senescence and all staygreen related traits with stress tolerance is supported by results showing that the same genomic regions have an effect on yield, grain weight, kernel number, canopy temperature, NDVI and also the length and rate of grainfilling. The staygreen character is clearly complex genetically with environmental influences that require further exploration.

### Author contribution statement

R Suzuky Pinto conducted field experiments, performed data analysis and led the write-up; Marta S. Lopes conducted field experiments, performed data analysis and provided useful advice for data interpretation; Nicholas C. Collins contributed to data interpretation and preparation of the manuscript; Matthew P. Reynolds designed the experiments and participated in all aspects of data analysis, interpretation and writing of the manuscript.

## Electronic supplementary material

Below is the link to the electronic supplementary material.

Supplementary material 1 (DOCX 21 kb)

Supplementary material 2 (DOCX 28 kb)

Supplementary material 3 (DOCX 21 kb)

Supplementary material 4 (DOCX 91 kb)

Supplementary material 5 (DOCX 47 kb)
