# Supplementary material for: Modelling and genetic dissection of staygreen under heat stress
Source: Theor Appl Genet. 2016 Aug 22;129(11):2055–74. doi: 10.1007/s00122-016-2757-4 (PMC5069319; doi:10.1007/s00122-016-2757-4)
Supplement: Supplementary file 1 — Supplementary material 1 (DOCX 21 kb) [file 122_2016_2757_MOESM1_ESM.docx]

Supplementary figure 1. PCA for Stg, TGW, KN and yield in the Seri/Babax population grown in M10, H05 and I13 heat-stressed, irrigated environments

TGW: thousand grain weight; KN: kernel number; Stg: residual greenness at physiological maturity; Environments: M10, moderate; H05, hot; I13, intense
