# Supplementary material for: Modelling and genetic dissection of staygreen under heat stress
Source: Theor Appl Genet. 2016 Aug 22;129(11):2055–74. doi: 10.1007/s00122-016-2757-4 (PMC5069319; doi:10.1007/s00122-016-2757-4)
Supplement: Supplementary file 3 — Supplementary material 3 (DOCX 21 kb) [file 122_2016_2757_MOESM3_ESM.docx]

Supplementary figure 3. Absolute rates of senescence (RS) for the two parents Seri and Babax grown under M10, H05 and I13 heat-stressed, irrigated environments

Bars indicate standard error
