# Supplementary material for: Modelling and genetic dissection of staygreen under heat stress
Source: Theor Appl Genet. 2016 Aug 22;129(11):2055–74. doi: 10.1007/s00122-016-2757-4 (PMC5069319; doi:10.1007/s00122-016-2757-4)
Supplement: Supplementary file 4 — Supplementary material 4 (DOCX 91 kb) [file 122_2016_2757_MOESM4_ESM.docx]

Supplementary figure 4. NDVIg patterns resulting after linking the dots of measurements performed on each of the two parents, Seri and Babax, in the M10, H05 and I13 heat-stressed, irrigated environments
