# Supplementary material for: Modelling and genetic dissection of staygreen under heat stress
Source: Theor Appl Genet. 2016 Aug 22;129(11):2055–74. doi: 10.1007/s00122-016-2757-4 (PMC5069319; doi:10.1007/s00122-016-2757-4)
Supplement: Supplementary file 5 — Supplementary material 5 (DOCX 47 kb) [file 122_2016_2757_MOESM5_ESM.docx]

Supplementary Table 1. Matrix of phenotypic correlations for all traits averaged across M10,

H05, H11, I06 and I13 heat-stressed, irrigated environments

Stg: staygreen at physiological maturity; RS: rate of senescence; TotalAUC: total area under the curve with starting points at crop establishment; StgAUC: staygreen area under the curve with starting points at maximum NDVI; Gdecay: percentage of greenness lost at mid grainfilling; KN: kernel number; TGW: thousand grain weight; GFR: grainfilling rate; GFD: grainfilling duration. NDVIv: normalized difference vegetative index during vegetative stage; NDVIg: normalized difference vegetative index during grainfilling; Chlv: chlorophyll content at vegetative stage (SPAD); Chlg: chlorophyll content at grainfilling (SPAD); CTv: canopy temperature at vegetative stage; CTg: canopy temperature at grainfilling.
